# Determination of the Kinetics and Thermodynamic Parameters of Lignocellulosic Biomass Subjected to the Torrefaction Process

**DOI:** 10.3390/ma14247877

**Published:** 2021-12-19

**Authors:** Maja Ivanovski, Aleksandra Petrovic, Irena Ban, Darko Goricanec, Danijela Urbancl

**Affiliations:** 1 Faculty of Chemistry and Chemical Engineering, University of Maribor, Smetanova Ulica 17, 2000 Maribor, Slovenia; maja.ivanovski@gmail.com (M.I.); aleksandra.petrovic@um.si (A.P.); irena.ban@um.si (I.B.); darko.goricanec@um.si (D.G.); 2 Department for Environment, Milan Vidmar Electric Power Research Institute, Hajdrihova Ulica 2, 1000 Ljubljana, Slovenia

**Keywords:** torrefaction, biomass, thermogravimetric analysis, kinetics, Friedman method, Kissinger–Akahira–Sunose method

## Abstract

The torrefaction process upgrades biomass characteristics and produces solid biofuels that are coal-like in their properties. Kinetics analysis is important for the determination of the appropriate torrefaction condition to obtain the best utilization possible. In this study, the kinetics (Friedman (FR) and Kissinger–Akahira–Sunose (KAS) isoconversional methods) of two final products of lignocellulosic feedstocks, miscanthus (*Miscanthus x giganteus)* and hops waste (*Humulus Lupulus*), were studied under different heating rates (10, 15, and 20 °C/min) using thermogravimetry (TGA) under air atmosphere as the main method to investigate. The results of proximate and ultimate analysis showed an increase in HHV values, carbon content, and fixed carbon content, followed by a decrease in the VM and O/C ratios for both torrefied biomasses, respectively. FTIR spectra confirmed the chemical changes during the torrefaction process, and they corresponded to the TGA results. The average *E_α_* for torrefied miscanthus increased with the conversion degree for both models (25–254 kJ/mol for FR and 47–239 kJ/mol for the KAS model). The same trend was noticed for the torrefied hops waste samples; the values were within the range of 14–224 kJ/mol and 60–221 kJ/mol for the FR and KAS models, respectively. Overall, the *E_a_* values for the torrefied biomass were much higher than for raw biomass, which was due to the different compositions of the torrefied material. Therefore, it can be concluded that both torrefied products can be used as a potential biofuel source.

## 1. Introduction

Under the Paris Agreement, which commits almost 200 countries to limiting climate change, the European Commission set a long-term goal for a climate-neutral Europe by 2050 [1]. The highest amount of energy is still produced by fossil fuels, which are, due to releasing greenhouse gases (GHG) and other air toxins/pollutants, causing severe environmental problems [2]. Without phasing out one of the main sources of greenhouse gas emissions, coal, the climate and energy goals set up in the European Green Deal [3] will be difficult to achieve. Recently, renewable and environmentally friendly energy sources have gained much attention in many applications [4], pointing out biomass as one of the most attractive ones.

Biomass is considered to be the most promising alternative to fossil fuel energy due to its specific characteristics [5], such as renewability, reproducibility, carbon neutrality, low cost, and abundant reserves [6,7]. Generally, biomass can be converted into three main product types: electrical/heat energy, transport fuel, and chemical feedstock to form solid, liquid, and gaseous products [8]. To obtain these products, thermochemical, biochemical, or physicochemical technological routes must be followed [9]. Furthermore, biomass is expected to play a significant role in the energy mix in the future, because its combustion produces clean energy by reducing greenhouse gas emissions [10,11]. As said, compared to other biomaterials it has a lot of advantages; however, disadvantages such as low calorific value, low energy density, and high moisture content may cause problems in its transport and/or storage [12]. To overcome these challenges and enhance the suitability of biomass as a potential solid biofuel source used in thermochemical processes, the torrefaction process is stepping up [13,14].

The torrefaction (or mild pyrolysis) process is defined as a pre-treatment method, where biomass is heated up at temperatures ranging from 200 °C to 300 °C, in atmospheric conditions and the absence of oxygen [15]. The process itself upgrades the fuel properties of biomass by reducing the moisture content, increasing the high heating value (HHV), increasing the carbon and oxygen content, and improving grindability. During the torrefaction process hydroxyl groups (–OH) are removed; therefore, torrefied materials are considered to be hydrophobic [16,17]. After torrefaction, biomass loses about 30% of the original mass, but retains 90% of the initial energy content [18]. Torrefied biomass is more attractive to the primary energy production sectors by promoting an increase in its fuel quality [19].

Numerous studies have been conducted to investigate the effects of the torrefaction process on raw biomaterials [20,21]. Some of them have reported how properties of torrefied biomaterials change with pressure [22,23], temperature and time [24,25], or atmosphere [26,27]. Dissimilar feedstocks such as wood chips [28,29], corn stalks [30,31], sewage sludge [32,33], and industrial wastes [34,35] have been applied, and followed by studying solid and energy yields, or calorific values. In all the above mentioned studies it was confirmed that the fuel properties of torrefied biomass were better than raw biomass. Additionally, Wang et al. [36], studied the effects of non-structural components (extractives and ash) on biomass characteristics. In their study, the materials were torrefied under a nitrogen atmosphere at 260 °C. The results indicated that the addition of organic extractives reduced deoxidation efficiency of structural components during torrefaction. The torrefaction process also leads to degradation of hemicelluloses and dehydration of cellulose and lignin [37]. According to this, Chen et al. [38] studied the properties of hemicellulose, cellulose, and lignin at a series of torrefaction temperatures (210, 240, 270, and 300 °C) based on the properties of their three-phase products (solid, liquid, and gaseous products). Results showed that, among the three biomass components, significant differences of torrefaction characteristics were found, due to their different molecular structures. Niu et al. [39] also confirmed that hemicelluloses have a primary effect on biomass property improvement.

Lately, the torrefaction mechanisms and thermal degradation behavior of biomass samples have been examined as well [40,41], conducted by torrefaction kinetics. As stated in the work of Castells et al. [42], it is important to understand the mechanisms that take place during the thermochemical processes and the kinetic parameters that define reactions.

Thermogravimetric analysis (TGA) is considered to be the most useful technique to define kinetics reactions for lignocellulosic biomass [28], as it is a rapid and inexpensive method for determining the physicochemical properties of biomasses, based on measuring the rate of weight loss as a function of temperature and time [43]. Two different types of methods can be applied from TGA to study kinetics mechanisms: model-fitting methods and model-free methods. Model-fitting methods fit different models to the obtained data, allowing the calculation of the apparent activation energy (*E_α_*) and pre-exponential factor (*A*). Model-free methods do not make any model assumptions, and determine activation energy (*E_α_*) as a function of the conversion factor or temperature. They are more complex, and more knowledge is needed to understand the reaction mechanisms. Friedman, Kissinger–Akahira–Sunose (KAS), and Flynn–Wall Ozawa (FWO) are common model-free methods for calculating kinetic parameters, and have been evaluated in many studies [44,45]. Doddapaneni et al. [25] studied the effect of torrefaction on kinetics, the reaction mechanism, and heat flow during the pyrolysis of biomass by making a comparative analysis between the pyrolysis of dried and torrefied eucalyptus wood. Kinetic analysis showed that torrefied biomass has higher *E_α_* than dried biomass. Chen et al. [46] studied the pyrolysis kinetics of rice husk, rice straw, and their products under different heating rates. Results showed that with increasing torrefaction temperature, the activation energy of rice husk and rice straw increased. Finally, Osman et al. (2019) [47] wrote a review of practical kinetic modeling approaches for the purpose to evaluate different processes for recycling, reusing, and upcycling biomass.

The goal of this work is to determine the kinetics parameters of the final products of two lignocellulosic biomasses, miscanthus and hops waste, using TGA analysis, and to select the most appropriate kinetic calculation method. Regardless of the numerous studies on biomass torrefaction, no study has yet been reported on the torrefaction process of hops waste as a raw biomaterial. Besides, thermodynamic parameters, like Gibbs free energy and change in enthalpy and entropy, were calculated using Eyring equations for the samples before and after the torrefaction process. It is believed that understanding the models applied and the final product characteristics are important for determining the appropriate torrefaction condition to obtain the best utilization.

The work is organized as follows: Materials, the torrefaction process, and kinetic theory are presented in Section 2; product characterization and kinetic parameters are discussed in Section 3; and finally, conclusions are exposed.

## 2. Materials and Methods

### 2.1. Materials

The samples studied in this work came from the Republic of Slovenia and were chosen as representatives of Slovenian biomass diversity and potential sources for thermochemical processes. The miscanthus (*Miscanthus x giganteus*) was collected in the region of Podravje, and was chosen for its energetic properties, which have been described by several authors [48,49], whereas the hops waste (*Humulus lupulus*) was collected in the region of Savinjska (Slovenia). Hops waste was chosen as one of the most commonly used agricultural plants in the country. It is a perennial bivalve plant grown mainly for the cones from which beer is produced, although some medical properties of it have already been discovered [50,51]. Hops waste was received with the white rope included (type TP 1000, UVS 1200). The white rope was used for holding the hops waste together.

Prior to the torrefaction process, both feedstocks were cut into similar sizes (15 × 3 × 5 and 25 × 5 × 2 mm^3^, respectively) to ensure homogeneity, and dried in a drier at 105 °C ± 2 °C until mass stabilization, according to the UNI EN 14774-1 protocol [52].

### 2.2. Torrefaction Process and Biomass Characterization

**Figure 1:**
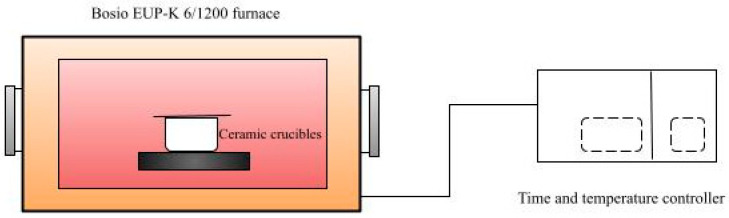
Experimental set-up of the torrefaction process.

**Figure 2:**
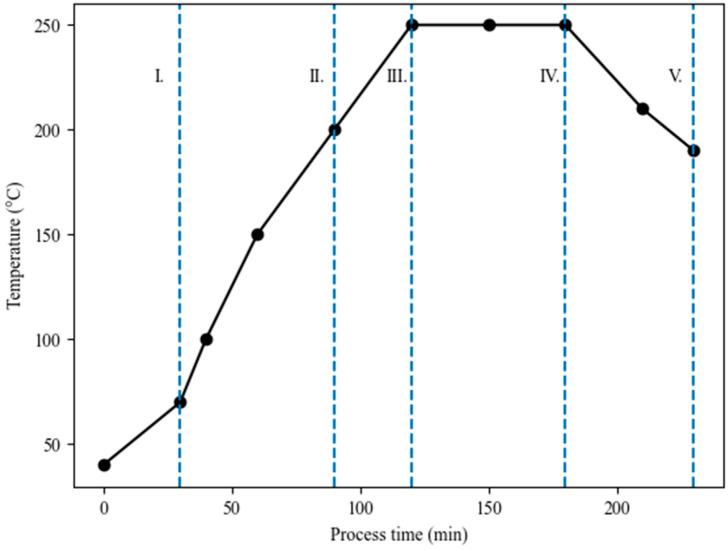
Torrefaction process in general.

In the present work, the torrefaction process (Figure 1) was performed in an electric lab-scale furnace, Bosio type EUP-K 6/1200 (Štore, Slovenia). The furnace consists of heating and regulating units. The nominal power was 2.7 kW. A detailed description of the Bosio industrial furnace is written in the work of Jóźwiak et al. [53]. The biomasses were torrefied at a temperature of 250 °C and residence time of 1 h. The torrefaction temperature was determined based on the obtained results for lignocellulosic biomass in our previous papers [14,54]. The torrefaction process was carried out in a semi-inert atmosphere generated by the lid, placed on the ceramic round crucibles specifically to create an inert atmosphere. The lid was placed in such a way that the flue gases, which had not been analyzed at this point of the research, and water vapor, could be removed. In our work, the torrefaction process followed the same heating stages as presented in Figure 2 ((heating (I), pre-drying (II), post-drying (III), torrefaction (IV), cooling (V)). After the torrefaction process, the biomasses were removed from the furnace, cooled down, and stored in hermetically sealed containers before being prepared for further analysis. Before each further analysis, the samples were again dried at 105 °C ± 2 °C for 1 h to a constant weight. The experiments were repeated three times to ensure measurement repeatability. The average values are used in the Discussion section.

The contents of the elements C, H, N, and S were determined by using a Perkin Elmer CHNS/O 2400 elemental analyzer (Billerica, MA, USA) following the international standards UNI EN 15104 and UNI EN 15289 [55,56], whereas the content of O was calculated by the difference, as presented in Equation (1).

O (%) = 100% − C (%) − H (%) − N (%) − S (%) − Ash (%)(1)

The moisture content (MC), volatile matter (VM), ash content (Ash), and fixed carbon (FC) content were determined in accordance with the ASTM D7582:2015 standard [57]. The high heating (HHV) and low heating values (LHV) were measured in an IKA Calorimeter C4000 adiabatic bomb calorimeter according to the UNI EN 14918 [58] and ISO 1928 DIN 51900 standards [59]. The mass and energy yields were calculated by the following equations (Equations (2) and (3)):

(2) $$\mathrm{Mass}\;\mathrm{yield}\;(\%)=\frac{{\;\mathrm{weight}}_{\mathrm{torrefied}}}{{\mathrm{weight}}_{\mathrm{raw}}}\;\times \;100$$

(3) $$\mathrm{Energy}\;\mathrm{yield}\;\left(\%\right)=\mathrm{Mass}\;\mathrm{yield}\;\times \;\frac{{\mathrm{HHV}}_{\mathrm{torrefied}}}{{\mathrm{HHV}}_{\mathrm{raw}}}$$

Both miscanthus and hops waste are lignocellulosic biomasses, which means they are composed of hemicelluloses, cellulose, and lignin. It is well-known that thermal degradation of hemicelluloses takes place between 200 and 320 °C, followed by cellulose decomposition between 300 and 360 °C, and lignin decomposition between 200 and 800 °C [60,61]. Decomposition of lignin is a slower process due to its complex chemical composition [62]. TAPPI standard T249 cm-85-2009 was used to determine the contents of the hemicelluloses and cellulose, whereas TAPPI standard T222 om-83-1988 was used to determine the content of lignin in this study. It was expected that the contents of hemicelluloses and cellulose would decrease, whereas the content of lignin would increase during the torrefaction, due to the three main reaction reactions that take place: decomposition, devolatilization, and depolymerization [9].

### 2.3. Fourier Transform–Infrared Spectroscopy (FTIR)

The raw and torrefied miscanthus and hops waste were characterized using Fourier transform–infrared spectroscopy (FTIR). The spectra were recorded within a wavelength that ranged between 4000 and 400 cm^−1^ using an FTIR spectrophotometer, Shimadzu IRAffinity (Tokyo, Japan). To perform the FTIR analysis, each of the dry samples was mixed with KBr (at a ratio of ~1:30) and pressed into tablet form.

### 2.4. Thermogravimetric Analysis

Thermogravimetric analysis (TGA) was performed using a Mettler Toledo TGA/SDTA instrument (Greifensee, Switzerland), type 851. The samples were heated from 30 °C to 800 °C, and the heating rate was set up at 10, 15, and 20 °C/min. In each round, between 8 mg and 10 mg of each sample were used. Prior to the analysis all samples were ground and sieved in order to obtain particle sizes lower than 1 mm, and later they were placed in aluminum crucibles. The experiments were replicated two times to verify repeatability. In this study, the thermogravimetric analyses were performed to evaluate the kinetics parameters of raw and torrefied samples exposed to thermal degradation under an air atmosphere. The weight losses of the samples were recorded, and the first derivative of the weight losses was determined during the TGA analyses.

### 2.5. Kinetic Theory

Osman et al. [47] stated in their work that understanding the thermal kinetics decomposition of biomass (biomass degradation) is crucial to identifying the physicochemical characteristics that hinder some of the energy generation applications, especially in lignocellulosic biomass samples. In this paper, the kinetic and thermodynamic parameters were determined using the model-free approaches based on integral isoconversional methods: the Friedman and Kissinger–Akahira–Sunose isoconversional methods. The choice of using these two methods was motivated by the fact that isoconversional methods do not require any assumption of the reaction models, which means that activation energy could be determined without pre-defining the reaction function; additionally, studying the complex solid-state processes is easier when applying these methods throughout the wide range of experimental conversions and temperatures set [63,64]. They also save time compared to the model-fitting methods [9]. The recommendations of kinetic modeling of the biomass thermal decomposition are well described and have been published by the ICTAC Kinetics Committee and other researchers [46,65,66,67].

The reaction conversion could be expressed as (Equation (4)):

(4) $$\frac{d\alpha }{dT}=k\left(T\right)\cdot f\left(\alpha \right)$$

where f(α) represents a function of the reaction model. *k*(*T*), the rate constant, is expressed by the Arrhenius Equation (5) below:

(5) $$k\left(T\right)=A\cdot e^{\left(-\frac{E_{\alpha }}{R\cdot T}\right)}$$

where *A* refers to a pre-exponential factor (min^−1^), *E_α_* to activation energy (kJ mol^−1^), *R* to a universal gas constant (8.314 J mol^−1^ K^−1^), and *T* to the absolute temperature (K^−1^). Furthermore, *f*(*α*) is assumed to be the first-order reaction (Equation (6)):

(6) f(α)=(1−α)

from this, α, the degree of conversion, can be determined as follows (Equation (7)):

(7) $$\alpha =\frac{m_{0}-m_{t}}{m_{0}-m_{f}}$$

where, *m*_0_, *m_t_*, and *m_f_*, are the initial, at any time, and final mass of the samples.

Giving Equation (5) into Equation (4):

(8) $$\frac{d\alpha }{dT}=A\cdot e^{\left(-\frac{E_{\alpha }}{R\cdot T}\right)}\cdot f\left(\alpha \right)$$

TGA analysis is carried out under constant heating rate $\beta =\frac{dT}{dt}$; therefore, the conversion can be expressed as the function of temperature [68]. For the reactions above it is proposed that the activation energy is independent of the temperature; therefore (Equation (9)):

(9) $$\frac{d\alpha }{dt}=\frac{d\alpha }{dT}\times \frac{dT}{dt}=\beta \frac{d\alpha }{dT}$$

From Equations (8) and (9):

(10) $$\frac{d\alpha }{dT}=\frac{A}{\beta }\cdot e^{\left(-\frac{E_{\alpha }}{R\cdot T}\right)}\cdot f\left(\alpha \right)$$

These expressions can be used to predict the solid reaction mechanism, reflected by the TGA curves. With integration of both sides of Equation (10):

(11) $$g\left(\alpha \right)=\int_{0}^{\alpha }\frac{d\alpha }{f\left(\alpha \right)}=\frac{A}{\beta }\cdot \int_{0}^{T}(e^{-\frac{E_{\alpha }}{R\cdot T}})\;dT$$

*G*(*α*) is the integral function of conversion degree α. The equations above are fundamental equations that are followed by appropriate kinetic methods to determine kinetics parameters during the thermochemical processes [69,70].

#### 2.5.1. Friedman Isoconversional Kinetic Model

Among isoconversional methods, the Friedman differential model (Table 1) is the most widely used model to evaluate the kinetics of biomass. The biggest advantage of this model is that it is not limited to using the linear variation of the heating rate [71]. It shows adequacy, accuracy, and simplicity. The Friedman isoconversional model has been studied by several researchers using various types of biomasses, like waste wood, waste straw, and sewage sludge, which have been studied by Sobek and Werle [72], or pine wood chips by Barzegar et al. [44].

#### 2.5.2. The Kissinger–Akahira–Sunose Isoconversional Kinetic Model

The Kissinger–Akahira–Sunose integral isoconversional model is a method where activation energy is assumed to be constant at a given conversion [73,74]. It has been applied to biomasses such as garlic hush, which was investigated by Sigh et al. [75], or kenaf (*Hibiscus cannabinus* L.), which was studied by Lee et al. [76]. The KAS method uses Murray and White’s approximation $p\left(x\right)=≅\frac{e^{x}}{x^{2}}$ for the temperature integral. The equation can be written as presented in Table 1.

### 2.6. Thermodynamic Parameters

The thermodynamic parameters, such as the pre-exponential factor (*A*), change of enthalpy (Δ*H*), entropy (Δ*S*), and Gibbs free energy (Δ*G*), were calculated using Eyring equations, which are presented by the equations in Table 2.

## 3. Results

### 3.1. Characterization of the Raw and Torrefied Material

**Figure 3:**
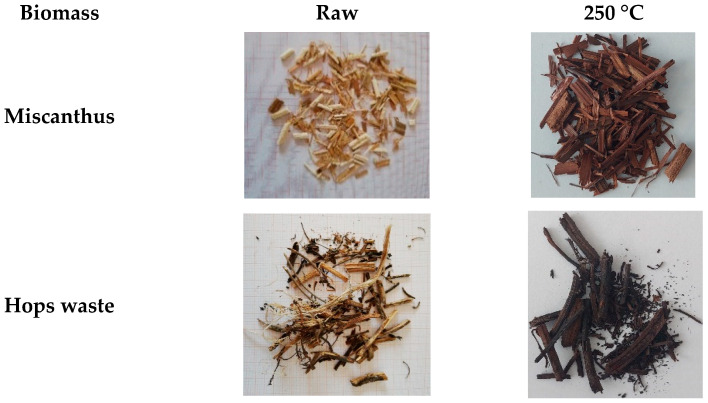
Raw and torrefied miscanthus and hops waste.

Figure 3 shows the biomass samples before and after the torrefaction process. The obtained torrefied samples presented a darker color than the raw biomass samples. This results from the fact that during the torrefaction process, as temperature increases, hydrocarbon molecules are broken down, and therefore, the carbon left is deposited on the surface of the sample.

**Figure 4:**
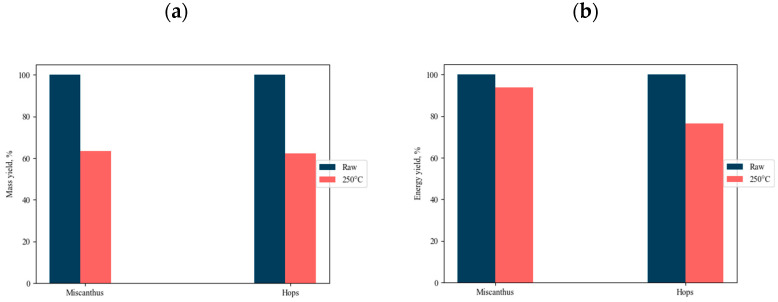
Mass yield (**a**) and energy yield (**b**) for both biomasses obtained during the torrefaction process.

The results of the ultimate and proximate analysis, mass, and energy yields of raw and torrefied miscanthus and hops waste at 250 °C and 1 h are shown in Table 3 and Figure 4. As observed, both mass and energy yields decreased when increasing the torrefaction temperature for both obtained biomaterials, respectively, which is in accordance with the literature [77,78]. Miscanthus is a perennial crop, known worldwide for its energetic properties and annual economy determination [79]. On the other hand, as already stated in this work, no torrefaction data on the hops waste have been published to date. Hops waste is a perennial dioecious plant, *H. lupulus*, grown mainly for the cones that are used in the brewing and pharmaceutical industries [80]. The leaves and stem materials are left unused and are transported to disposal sites for burning [81]. Increasing the temperature leads to increasing the fixed carbon content and decreasing volatile matter. The fixed carbon content for torrefied miscanthus increased from 3.9 wt.% to 14.9 wt.% and from 1.9 wt.% to 7.5 wt.% for torrefied hops waste. A decline in the content of volatile matter was observed in both biomasses, which may have had an additional an impact on the calorific values (HHV) in the biomasses. The higher the content of volatile matter is, the lower is the calorific value in torrefied biomass and the more reactive the fuel. On the contrary, the higher the fixed carbon content is, the higher the calorific value of the torrefied biomass is and the less reactive the fuel is [10]. The HHV of miscanthus was measured to be 16.4 MJ/kg for the raw and 21.1 MJ/kg for the torrefied samples, whereas the HHV of hops waste was 16.5 MJ/kg and 18.9 MJ/kg for a raw sample and torrefied sample, respectively. The ash content for both biomaterials increased when torrefied at 250 °C, and was 5.75% and 6.687%, respectively. It is believed that that kind of increase in ash content is due to the weight loss of volatile matters that are released during the process. Besides, the higher the torrefaction temperature is, the greater the volatile release is. Kaur et al. [82] stated that there are several disadvantages of high ash content presented in the biomaterial. Those include high processing costs, poor combustion, reduced energy conversion, and disposal problems. Furthermore, the torrefaction process leads to enrichment of the carbon content, whereas the oxygen and hydrogen content decreases. The C content increased from 46.2 wt.% to 55.5 wt.% for torrefied miscanthus, and from 42.4 wt.% to 48.6 wt.% for torrefied hops waste. On the contrary, the H and O contents decreased in both torrefied samples. For miscanthus the H content declined from 4.1 wt.% to 3.9 wt.%, and for O from 48.9 wt.% to 39.9 wt.%, whereas for hops waste the contents decreased from 4.9 wt.% to 3.4 wt.% and from 36.9 wt.% to 24.2 wt.%, respectively. As expected, the contents of nitrogen and sulfur were low. Similar variations have already been reported elsewhere for miscanthus [83,84]: Both the O/C and H/C ratios decreased as torrefaction temperature increased. This is due to moisture and volatile removal from the samples that contain more hydrogen and oxygen content than carbon content. From this, it can be concluded that the torrefaction temperature really affects the chemical components of the investigated biomass, whereas residence time does not have as much of an impact on chemical components as temperature.

### 3.2. Fourier Transform–Infrared Spectroscopy (FTIR)

**Figure 5:**
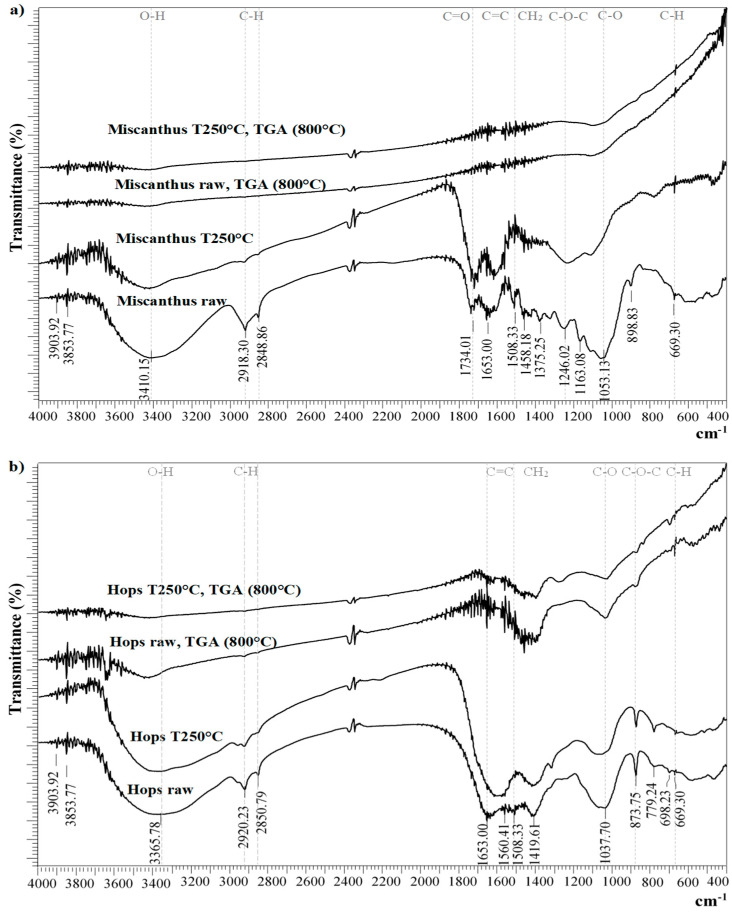
FTIR spectra for miscanthus (**a**) and hops waste (**b**) samples exposed to different thermal treatment procedures.

The FTIR spectra of miscanthus and hops waste samples are shown in Figure 5a,b. The spectra of raw materials show peaks typical for lignocellulosic materials. The absorption bands between 2800 and 3000 cm^−1^ correspond to the C–H stretching vibration in the aliphatic and aromatic compounds [85], whereas the wide peak from 3600 to 3200 cm^−1^ corresponds to the vibrations of the hydroxyl groups (–OH) of the cellulose [86]. The stretching vibrations of C–H and CH_2_ groups also appeared in the area between 1300 and 1460 cm^−1^. The peaks at 1508 and 1653 cm^−1^ could be assigned to aromatic C=C stretching in the lignin [87]. The vibrations of the C–O group, typical for cellulose, were found at around 1050 cm^−1^. The weak peaks at around 779 and 669 cm^−1^ could be associated with the presence of aromatic hydrogen [88].

In the miscanthus sample some additional peaks were found, for example, a peak at 1734 cm^−1^ related to the C=O vibrations, and peaks at 1246, 1163, and 899 cm^−1^ associated with C-O-C linkages between the hemicellulose or lignin [89].

After torrefaction, the intensity of some smaller peaks in the area between 1000 and 1500 cm^−1^ and those at around 2900 cm^−1^ dropped in both torrefied samples. This was connected with the releasing of volatile compounds from the biomass as a consequence of low-temperature thermic treatment, during which the hemicellulose and lignin were partly decomposed and lost their structures. In addition, some peaks disappeared, whereas others shifted or became stronger, such as the peaks for C–C and C–O stretching. High-temperature treatment of the samples (800 °C) resulted in the disappearance of the majority of the peaks representing characteristic functional groups for cellulose, hemicellulose and lignin, since these compounds were almost completely degraded in both samples. Among several disappeared bands was, for instance, the band for the hydroxyl groups of cellulose (3200–3600 cm^−1^). However, a significant difference was observed between the miscanthus and hops waste samples after treatment at 800 °C: The intensity of the peaks at 1458, 1034, 878, and 669 cm^−1^ for the hops waste samples was much stronger, which could most likely be connected to the differences in the basic composition of these two materials, which, consequently, impacted the composition of the final products.

According to the FTIR spectra, both miscanthus and hops waste were affected by similar chemical changes during the torrefaction process. The FTIR spectra of raw and torrefied miscanthus were in agreement with those found in one of the previous studies [90], whereas for torrefied hops waste there is a lack of data in the literature.

### 3.3. The Thermal Degradation Process

**Figure 6:**
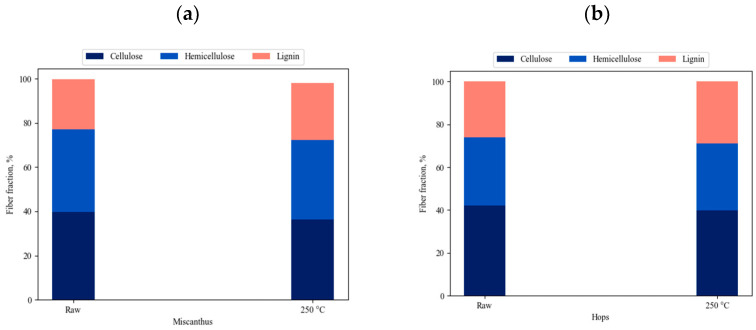
Contents of cellulose, hemicellulose, and lignin in raw and torrefied miscanthus (**a**) and hops waste (**b**).

The weight-loss curves obtained during the TGA analyses under an air atmosphere are presented below. The samples were heated from room temperature, 25 °C, to 800 °C, at three different heating rates of 10, 15, and 20 °C/min. The decomposition of hemicelluloses, cellulose, and lignin were observed on the TGA and DTG curves. Figure 6 shows the contents of all three compounds in miscanthus and hops waste before and after the torrefaction process. Generally, the decomposition of hemicelluloses, cellulose, and a small amount of lignin appeared in the main stage of combustion, at a temperature range of 180–550 °C, where the highest weight-loss rate was observed for all the analyzed samples. The mass started to decrease at about 300 °C, which was in accordance with the temperature range reported for the decomposition of hemicellulose that occurs at 220–315 °C, and cellulose that occurs at 315–400 °C [44,91]. It has also been reported that the cell walls of miscanthus contain 30–44% cellulose, 29–42% hemicellulose, 7–21% lignin, and a very small amount of ash [92], which is in agreement with our results.

**Figure 7:**
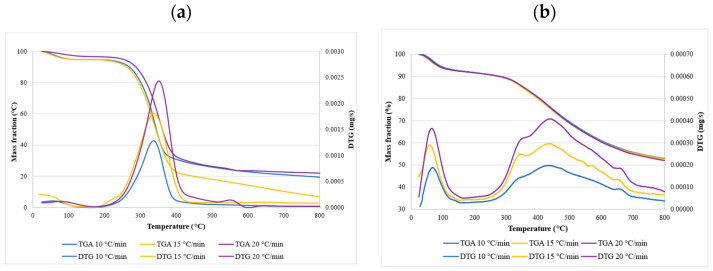
Thermogravimetric curves and derivative thermogravimetric curves for raw miscanthus (**a**) and for torrefied miscanthus at 250 °C (**b**).

**Figure 8:**
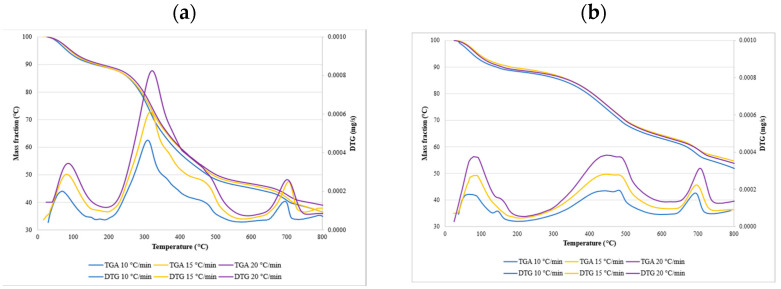
Thermogravimetric curves and derivative thermogravimetric curves for hops waste raw (**a**) and for hops waste torrefied at 250 °C (**b**).

Figure 7 and Figure 8 show the TGA and DTG curves for both samples. The TGA profiles of the chosen samples recorded under an air atmosphere at first sight looked very similar, whereas the DTG profiles revealed more significant differences. Generally, the decomposition process can be divided into three stages. The release of weakly bonded water molecules and hydrolysis was observed in the first stage. This stage is known as the evaporation or dehydration stage. The DTG curves of raw and torrefied miscanthus and torrefied hops waste showed a significant peak around 100 °C, corresponding to the moisture evaporation phase. That peak was much smaller in raw miscanthus, due to the very low moisture content. An initial drop in the mass curves could be seen at the beginning, and ended at about 100 to 150 °C. The weight loss for miscanthus samples was, in this stage, around 6%, and for hops waste samples around 10%. On the other hand, the DTG peak of hemicellulose became smaller for torrefied materials, which confirmed the large loss of hemicellulose content during torrefaction. Similar observations were found in the study performed on corn cob torrefaction [8]. However, the weight of torrefied samples in the second stage decreased by approx. 25–30%, whereas in raw samples, it decreased by approximately 45–50%. The last stage occurred at temperatures higher than 550 °C, and was connected mainly to the lignin decomposition and combustion of complex organic components. The hops waste samples also showed a significant peak at around 700 °C, which could be connected to the decomposition of some inorganic materials such as carbonate for its endothermic process [93]. As could be seen, torrefaction had a huge impact on the overall weight loss of miscanthus samples, since the overall weight loss of torrefied samples was about 30% lower than the weight loss of the raw samples. For hops waste samples that difference was lower, around 15–18% (Table 4). In addition, the weight loss of miscanthus was higher than the weight loss of hops waste, reflecting the significant differences in their basic compositions.

The TGA curves showed that torrefied materials have an initial decomposition temperature higher than raw materials, since torrefaction reduces the thermal stability of cellulose, resulting in an increase in initial temperature. The information about the heating rate is necessary, as it influences the conversion. From the DTG curves it can be observed that higher heating rates shifted the peak temperature (Table 4) to a higher value. The change in behavior was due to poor heat transfer. Lower heating rates are usually preferred, as the heating of the biomass particles is constant and allows better heat transfer to the interior of the biomass.

### 3.4. Kinetic Analysis

The kinetics parameters for miscanthus and hops waste samples before and after the torrefaction process at 250 °C were determined using the FR method and KAS method, which, in the past, were proven to be the most appropriate methods to describe the kinetic behavior of lignocellulosic biomasses [94]. Thermogravimetric data obtained from the TGA curves recorded in air atmosphere at 10, 15, and 20 °C/min were used to calculate the apparent activation energies (*E_α_*) and pre-exponential factors (*A*) at each conversion degree (α). Data for raw samples are shown for conversion values α between 0.1 and 0.8, whereas the range of 0.1–0.7 was used for torrefied samples. Below or above that, the values of the correlation coefficients were very low, which was probably due to the thermal behavior of lignin [95], and therefore they are not included in the Discussion section.

#### 3.4.1. Activation Energy

**Figure 9:**
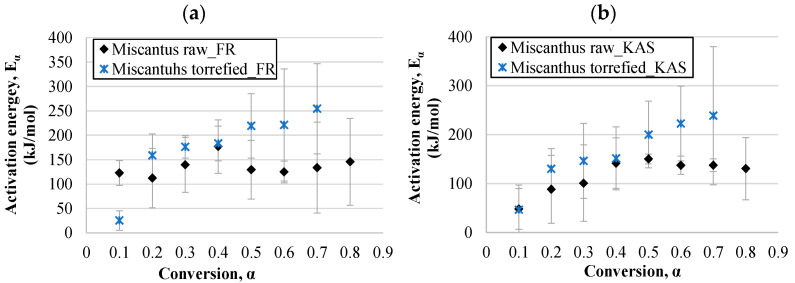
Activation energies dependent on the conversion degree for miscanthus samples calculated by the Friedman (**a**) and KAS (**b**) models.

**Figure 10:**
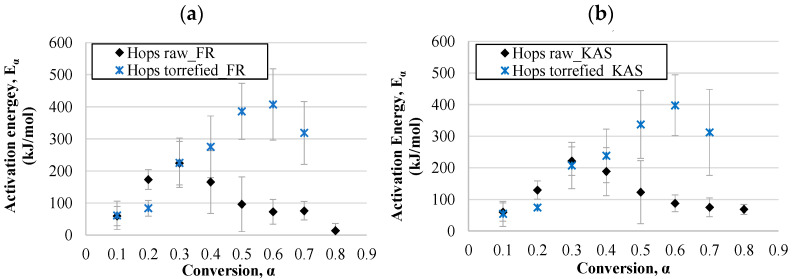
Activation energies dependent on the conversion degree for hops waste samples calculated by the Friedman (**a**) and KAS (**b**) models.

The activation energy represents the minimum amount of energy that is required to start a reaction; therefore, knowledge of activation energy data is necessary for the evaluation of the energy potential of biomass. The values of activation energies determined on the basis of isoconversional linear plots (using the Equations in Table 1) with respect to the conversion degree for miscanthus samples are shown in Figure 9, and for hops waste samples in Figure 10. In this study, the values of activation energies for raw miscanthus in the FR model varied between 112 and 177 kJ/mol (average value of 136 kJ/mol), wherein, in principle, they increased at lower conversion degrees. At the conversion degree of 0.5 a drop was observed, and then at the highest conversions they increased again. The KAS model gave values in a wider range, 48–150 kJ/mol, with the trend of increasing up to a conversion of 0.5, and afterwards the values were relatively stable, or even decreased. The increase in activation energies with regard to the increase in torrefaction temperature may have been due to the hemicellulose depletion during the torrefaction process. At the beginning, hemicellulose started to degrade at very low temperatures, for which low activations were required. For the torrefied miscanthus sample the activation energies increased with the conversion degree all the time for both models, and the results of both models were closer (25–254 kJ/mol for the Friedman model and 47–239 kJ/mol for the KAS model). The same trend was noticed for the torrefied hops waste samples, with an exception at the highest conversion (0.7), where the activation energy dropped significantly. This may have been due to the specific biomass structure and lignin degradation that occurred at this stage of the process. However, it must be considered that the correlation coefficient for that point was relatively low. Since the torrefaction process increases the stability of biomass, the activation energies increased in the case of torrefied samples. The same was confirmed by Tian et al. [8] for corncob samples. In addition, Wilk et al. [96] worked with three different feedstocks, miscanthus, pine, and acacia, to determine their kinetic parameters. The authors stated that the torrefaction process led to a decrease in *E_α_* compared to raw biomass. The *E_α_* values of miscanthus may also be explained to some extent by the dependence of its chemical and pore structure change on temperature. The comparison of torrefied miscanthus and hops waste samples revealed that for the decomposition of torrefied hops waste, almost double the amount of activation energy is needed, as the activation energies for torrefied hops were in the range of 61–407 kJ/mol in the case of the Friedman model, and between 54 and 398 kJ/mol for the KAS model. A similar difference was observed for the raw hops waste and raw miscanthus samples, since the activation energies of the latter were also lower, indicating that degradation of raw hops waste is more complex than that of miscanthus. Otherwise, the activation energies for raw hops increased up to a conversion degree of 0.3, and they decreased with further increase in the conversion. The values were within the range of 14–224 kJ/mol and 60–221 kJ/mol for the Friedman and KAS models, respectively. The literature survey showed that the average activation energies for raw miscanthus reported in the literature are between 78 and 212 kJ/mol (determined by several kinetics models, including the KAS/FR methods) [97,98,99], which is close to the values achieved in this study. For instance, the activation energies reported by Cortés and Bridgwater [100] for raw and torrefied miscanthus were in the range of 129 to 156 kJ/mol using the same isoconversional models. A comparison for torrefied hops waste samples cannot be made, as data could not be found on the kinetics or thermodynamics of the thermic degradation of torrefied hops waste.

Hence, when comparing the kinetic models, the Friedman model principally provided values of activation energies in a wider range, and the highest activation energies for all the tested samples were calculated by the Friedman method. However, the correlation coefficients of both were relatively close. The differences in activation energies between the models used comes from the different approximations and calculations used to solve the temperature integral when applying the model-free method. The activation energy is also proportional to the stability of the material, and is also used to identify the reactivity of a fuel [101]. According to the literature, the activation energies of hemicelluloses, cellulose, and lignin are in the range of 90–125 (Stage I), 145–285 (Stage II), and 30–39 kJ/mol (Stage III) [82]. The values obtained in this study likewise varied strongly with the conversion, which is closely connected to the degradation of the abovementioned components at a certain conversion degree.

#### 3.4.2. The Pre-Exponential Factor

The pre-exponential factor, *A*, is the constant in an Arrhenius equation, and describes the relationship between the temperature and the reaction rate constant [102,103]. The obtained pre-exponential factors are presented in Table 5 and Table 6 for miscanthus and hops waste, respectively. The values of pre-exponential factors for the miscanthus raw sample ranged from 2.59 × 10^07^ to 1.38 × 10^13^ using the Friedman method, and between 3.5 × 10^01^ and 6.1 × 10^10^ for the KAS method. For the torrefied miscanthus sample, the range was wider, as the values increased, for example, up to 5.25 × 10^16^ when applying the Friedman model (KAS–3.46 × 10^15^). Similar values applied to the hops waste samples, except that the values were much higher. For the torrefied hops waste sample, values up to 2.42 × 10^26^ were calculated with the Friedman method, and up to 5.10 × 10^26^ with the KAS method. Thus, both torrefied samples gave higher pre-exponential factors than their corresponding raw samples. The above values also confirmed that decomposition of the torrefied hops waste sample is more complex. The values for the miscanthus sample are in agreement with those in the literature [97,100]. Likewise, for hops waste samples no experimental data have been found yet.

#### 3.4.3. Kinetic Compensation Effect

**Figure 11:**
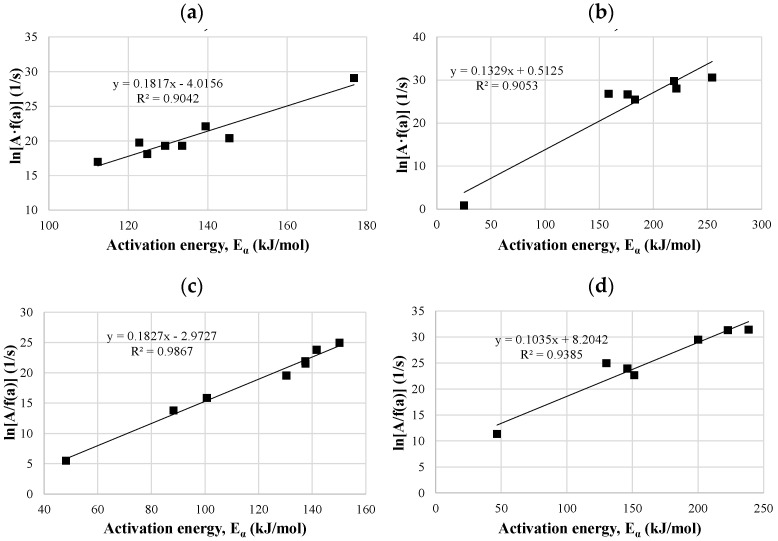
Compensation plots of pre-exponential factors versus E_α_ for the miscanthus raw sample calculated by the Friedman (**a**) and KAS (**c**) methods, and for the miscanthus torrefied sample calculated by the Friedman (**b**) and KAS (**d**) methods.

**Figure 12:**
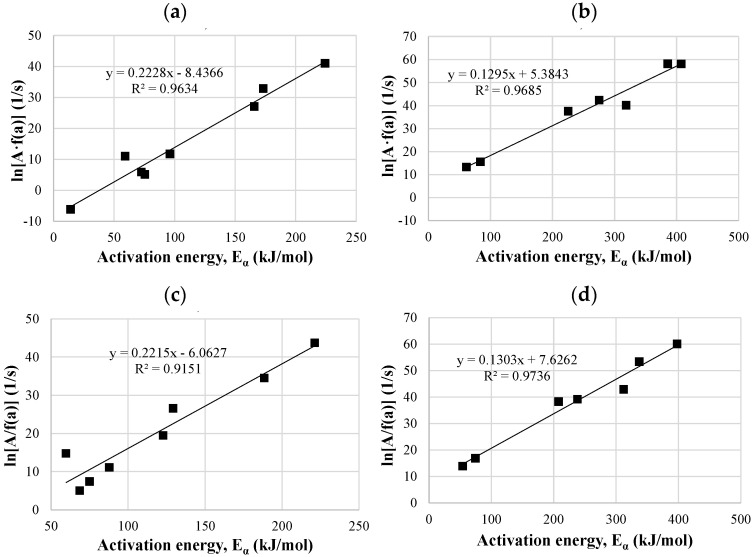
Compensation plots of pre-exponential factors versus E_α_ for the hops waste raw sample calculated by the Friedman (**a**) and KAS (**c**) methods, and for the hops waste torrefied sample calculated by the Friedman (**b**) and KAS (**d**) methods.

The relation between pre-exponential factors and activation energy could be presented by the kinetic compensation effect. In the thermochemical conversional processes, the kinetic compensation effect has been studied extensively [25,104]. As can be seen from Figure 11 and Figure 12, both biomasses showed a linear relationship between pre-exponential factors and activation energy for all the tested samples, which confirms the existence of the compensation effect between these two parameters. Both kinetic models, KAS and Friedman, gave the correlation coefficients for compensation plots >0.90, wherein the KAS model exhibited slightly higher values for miscanthus samples, and Friedman for hops waste samples. Thus, both models proved to be suitable for the analysis of the obtained thermogravimetric data.

### 3.5. Thermodynamic Parameters

The thermodynamic parameters of enthalpy (Δ*H*), entropy (Δ*S*), and Gibbs free energy (Δ*G*) for the miscanthus samples and hops waste samples are shown in Table 5 and Table 6. The values were calculated at DTG peak temperatures.

#### 3.5.1. Enthalpy (Δ*H*)

The variation in enthalpy follows a similar trend as the activation energy. The enthalpy (Δ*H*) values calculated by the Friedman method for the miscanthus raw sample were between 107 and 172 kJ/mol, whereas for the torrefied sample between 19 and 248 kJ/mol the KAS model gave similar values as the Friedman model. The values obtained for the miscanthus samples were in agreement with those found in the work of Wilk et al. [96]. For the raw hops waste sample, Δ*H* values resulted from 9 to 219 kJ/mol and from 55 to 216 kJ/mol separately for the Friedman and KAS methods, respectively. For the torrefied hops waste sample values were between 55 and 401 kJ/mol and 48 and 391 kJ/mol, respectively.

The difference between the values of activation energy and enthalpy for the miscanthus raw sample at each conversion point was 5.1 kJ/mol, and for the torrefied sample 5.9 kJ/mol. For raw hops waste and torrefied hops waste these values were equal to 4.8 and 6.3 kJ/mol, respectively. According to the results obtained, more heat energy is required to dissociate the bonds in torrefied samples than in raw samples.

#### 3.5.2. Entropy (Δ*S*)

The changes in entropy (Δ*S*) for the miscanthus raw sample were negative at all conversion degrees, as Δ*S* varied from −117 to −8 kJ/mol for the Friedman method and from −230 to −53 kJ/mol for the KAS method. On the other hand, for the torrefied miscanthus sample and hops waste samples, both negative and positive values were characteristic, which reflects the complexity of the thermic degradation of those samples into final products. For raw hops waste, sample entropy values ranged between −294 and 91 kJ/mol, and −203 and 85 kJ/mol, separately for the Friedman and KAS methods, respectively. For torrefied hops waste a wider range was noticed, from −231 to 244 kJ/mol for the Friedman and from −241 to 231 kJ/mol for the KAS model. The low Δ*S* means that the product was near to its thermodynamic equilibrium state and the material showed low reactivity, whereas a high Δ*S* means high reactivity, and less time was consumed to form an activated complex.

#### 3.5.3. Gibbs Free Energy (Δ*G*)

Gibbs free energy (Δ*G*) varied from 176 to 179 kJ/mol and from 177 to 183 kJ/mol for the miscanthus raw sample for both methods; for torrefied miscanthus it varied from 206 to 220 kJ/mol and from 207 to 216 kJ/mol. Similar to the case of the miscanthus samples, torrefied hops waste likewise showed higher values than the corresponding raw material—they varied between 217 and 229 kJ/mol for the Friedman method, and 217 and 230 kJ/mol for the KAS method. For the raw hops waste sample, Δ*G* was in the range between 167 and 180 kJ/mol for the Friedman and 170 and 173 kJ/mol for the KAS method. As can be seen, the Gibbs free energy was relatively stable in all the tested samples, showing only slight variations between the upper and lower limits. The values of the kinetic models used were comparable. Gibbs free energy indicates the total increase in energy of the system for the formation of the activated complex, and reflects the bioenergy potential of the biomass. According to the obtained results the analyzed feedstocks have decent bioenergy potential.

## 4. Conclusions

Miscanthus and hops waste were studied to evaluate their potential for biofuel production by torrefaction. Both samples were torrefied under a semi-inert atmosphere at 250 °C for 1 h. We can conclude that the torrefaction process led to an increase in carbon content and calorific value of up to 28% for miscanthus, and up to 15% for hops waste. According to the FTIR spectra, both samples had similar properties, but the process biodegradable substances were decomposed after torrefaction. Therefore, it can be stated that a stable biomass with higher calorific value was obtained during the process. Moreover, the TGA profiles showed that torrefaction had a great influence on the weight loss of both biomass samples during thermal degradation. The peak temperature of the DTG curves was higher in the case of the torrefied materials. The data from the TGA analyses were later used to evaluate the kinetics and thermodynamic properties of the biomass samples using two isoconversion methods, KAS and the Friedman method. Both methods proved to be suitable for the kinetic analysis of the obtained data, and showed comparable results. The values of the activation energies of the torrefied samples, which were higher than those of the raw samples, along with the values of the thermodynamic parameters, reflect the complexity of the thermal degradation of the torrefied materials.

## Figures and Tables

**Table 1:** Kinetics expressions for specific methods.

Method	Kinetic Expression
Friedman method (FR)	$\ln\beta \;\left(\frac{d\alpha }{dT}\right)=\ln\left[f\left(\alpha \right)\cdot A\right]-\frac{E_{\alpha }}{R\cdot T}$
Kissinger–Akahira–Sunose (KAS)	$\ln\left(\frac{\beta }{T^{2}}\right)=\ln\left[\frac{A\cdot R}{E_{\alpha }\cdot g\left(\alpha \right)}\right]-\frac{E_{\alpha }}{R\cdot T}$

**Table 2:** Calculation of thermodynamic parameters.

Method	Kinetic Expression
Pre-exponential factor (*A*)	$A=\frac{\beta \cdot E_{\alpha }\cdot e^{\left(-\frac{E_{\alpha }}{R\cdot T_{p}}\right)}}{R\cdot T_{p}^{2}}$
Change of enthalpy (Δ*H)*	$\Delta H=E_{\alpha }-R\cdot T$
Entropy (Δ*S*)	$\Delta G=E_{\alpha }+R\cdot T_{p}\cdot ln\left(\frac{K_{b}\cdot T_{p}}{h\cdot A}\right)$
Gibbs free energy (Δ*G*)	$\Delta S=\frac{\Delta H-\Delta G}{T_{p}}$

*K_b_* (Boltzman constant) =1.381 × 10^−23^ m^2^kg/s^2^ K; h (Plank constant) = 6.626 × 10^−34^ m^2^kg/s; *T_p_* is the peak temperature of the DTG curve.

**Table 3:** Elemental and proximate analysis of raw and torrefied miscanthus and hops waste.

Material	Torrefaction Temperature (°C)	Elemental Analysis (wt.%, Dry Basis)							MC (wt.%, Dry Basis)	Proximate Analysis (wt.%, Dry Basis)			HHV (MJ/kg)
		**C**	**H**	**N**	**O**	**S**	**H/C**	**O/C**		**FC**	**VM**	**Ash**	
Miscanthus raw	/	46.2	4.1	0.8	48.9	0.03	1.0	0.8	9.3	3.9	83.9	2.9	16.4
Miscanthus torrefied	250	55.5	3.9	0.6	39.9	0.01	0.9	0.5	0	14.6	72.4	5.8	21.1
Hops waste raw	/	42.4	4.9	2.5	36.9	0.02	1.4	0.7	11.8	1.9	82.9	3.4	16.5
Hops waste torrefied	250	48.6	3.4	3.1	24.2	0.01	0.8	0.4	0	7.5	74.8	6.7	18.9

**Table 4:** The peak temperatures (Tp) of DTG curves and weight loss of samples at the tested heating rates: 10, 15, 20 °C/min^−1^.

Sample		10 °C min^−1^		15 °C min^−1^		20 °C min^−1^	
		Tp	Weight Loss (wt. %)	Tp	Weight Loss (wt. %)	Tp	Weight Loss (wt. %)
Miscanthus	Raw	336.2	80.5	337.8	82.3	351.5	77.9
	Torrefied at 250 °C	436.8	47.1	434.2	47.3	440.6	48.0
Hops waste	Raw	308.8	63.5	317.0	63.5	321.7	61.1
	Torrefied at 250 °C	480.6	48.2	443.3	45.3	448.5	46.2

**Table 5:** Kinetic and thermodynamic parameters (*A*, Δ*H*, Δ*G*, Δ*S*) calculated by the Friedman and KAS models for miscanthus raw and torrefied samples exposed to TGA under air atmosphere.

Conversion Degree, α	Friedman Method					KAS Method				
	R^2^	A (1/s)	Δ*H* (kJ/mol)	Δ*G* (kJ/mol)	Δ*S* (J/mol·K)	R^2^	*A* (1/s)	Δ*H* (kJ/mol)	Δ*G* (kJ/mol)	Δ*S* (J/mol·K)
Miscanthus raw										
0.1	0.97	2.22 × 10^8^	117.67	178.22	−99.39	0.63	3.50 × 10	43.08	182.96	−229.62
0.2	0.76	2.59 × 10^7^	107.24	178.67	−117.26	0.67	1.75 × 10^5^	83.17	179.89	−158.78
0.3	0.89	6.83 × 10^9^	134.39	177.57	−70.90	0.68	2.36 × 10^6^	95.67	179.22	−137.15
0.4	0.93	1.38 × 10^13^	171.74	176.37	−7.60	0.91	1.07 × 10^10^	136.59	177.50	−67.14
0.5	0.85	8.46 × 10^8^	124.19	177.96	−88.26	1.00	6.10 × 10^10^	145.10	177.20	−52.69
0.6	0.98	3.36 × 10^8^	119.69	178.14	−95.95	0.99	4.50 × 10^9^	132.35	177.65	−74.36
0.7	0.72	2.03 × 10^9^	128.46	177.79	−80.98	1.00	4.63 × 10^9^	132.48	177.64	−74.13
0.8	0.34	2.32 × 10^2^	140.37	177.36	−60.72	0.84	1.06 × 10^9^	125.29	177.92	−86.39
Miscanthus torrefied (250 °C)										
0.1	0.56	7.25 × 10^−2^	19.35	219.70	−282.26	0.53	5.07	40.79	216.08	−246.94
0.2	0.94	3.02 × 10^9^	152.81	208.86	−78.96	0.93	1.93 × 10^7^	124.15	210.03	−120.99
0.3	0.72	6.58 × 10^10^	170.38	208.24	−53.33	0.82	3.29 × 10^8^	140.21	209.34	−97.39
0.4	0.70	2.19 × 10^11^	177.25	208.01	−43.33	0.62	8.30 × 10^8^	145.47	209.14	−89.70
0.5	0.69	1.15 × 10^14^	213.15	206.95	8.73	0.64	4.19 × 10^12^	194.14	207.49	−18.80
0.6	0.83	1.68 × 10^14^	215.32	206.90	11.87	0.82	2.21 × 10^14^	216.91	206.85	14.17
0.7	0.58	5.25 × 10^16^	248.42	206.07	59.65	0.56	3.46 × 10^15^	232.74	206.45	37.05

**Table 6:** Kinetic and thermodynamic parameters (*A*, Δ*H*, Δ*G*, Δ*S*) calculated with the Friedman and KAS models for hops waste raw and torrefied samples to TGA under air atmosphere.

Conversion Degree, α	Friedman Method					KAS Method				
	R^2^	*A* (1/s)	Δ*H* (kJ/mol)	Δ*G* (kJ/mol)	Δ*S* (J/mol·K)	R^2^	*A* (1/s)	Δ*H* (kJ/mol)	Δ*G* (kJ/mol)	Δ*S* (J/mol·K)
Hops waste raw										
0.1	0.83	7.22 × 10^2^	54.34	173.06	−204.07	0.85	8.22 × 10^2^	54.92	173.01	−202.98
0.2	0.98	3.56 × 10^13^	168.24	167.87	0.64	0.96	3.12 × 10^9^	124.46	169.28	−77.03
0.3	0.93	1.79 × 10^18^	219.33	166.61	90.62	0.97	9.41 × 10^17^	216.30	166.68	85.30
0.4	0.78	7.32 × 10^12^	160.80	168.08	−12.51	0.89	9.27 × 10^14^	183.60	167.45	27.74
0.5	0.38	2.44 × 10^6^	91.29	170.71	−136.52	0.66	7.62 × 10^8^	117.90	169.53	−88.75
0.6	0.82	1.38 × 10^4^	67.64	172.08	−179.52	0.93	3.99 × 10^5^	82.97	171.15	−151.56
0.7	0.90	2.66 × 10^4^	70.62	171.88	−174.07	0.89	2.42 × 10^4^	70.18	171.91	−174.87
0.8	0.35	1.54 × 10^−2^	9.25	180.00	−293.50	0.96	5.85 × 10^3^	63.75	172.34	−186.67
Hops waste torrefied (250 °C)										
0.1	0.71	3.80 × 10	54.97	228.82	−230.69	0.71	1.05 × 10	47.72	229.61	−241.36
0.2	0.94	1.86 × 10^3^	77.38	226.86	−198.37	0.99	3.85 × 10^2^	68.24	227.59	−211.45
0.3	0.85	3.22 × 10^13^	218.90	220.66	−2.34	0.91	1.78 × 10^12^	201.28	221.17	−26.40
0.4	0.88	1.13 × 10^17^	268.78	219.40	65.52	0.87	2.64 × 10^14^	231.73	220.31	15.16
0.5	0.56	7.34 × 10^24^	379.37	217.29	215.09	0.56	2.73 × 10^21^	330.74	218.13	149.43
0.6	0.69	2.42 × 10^26^	400.93	216.95	244.15	0.70	5.10 × 10^25^	391.32	217.10	231.20
0.7	0.43	1.33 × 10^20^	312.17	218.49	124.32	0.52	4.63 × 10^19^	305.76	218.62	115.53
